# The Title of Professor

**Published:** 1894-10

**Authors:** 


### The Title of Professor.

In German universities the teaching body is divided into three classes. The aspirant to a professorship receives the *venia legendi* under the name of *Privatdocent.* Above him is the professor extraordinarius, and on the highest rung of the ladder there is the professor ordinarius, who is eligible for the deanship of his faculty and for the rectorship of the university. The title of professor being a much coveted one in Germany, the government has from time to time bestowed it on men of special merit; either *Privatdocenten*, for whom no professorship could at this moment be found, or other distinguished men not connected with the university. This government favor, by the way, has the additional merit of cheapness, as in such cases the title is purely honorary, no remuneration being attached to it. Of late, quite a shower of these titular professorships has fallen. The Prussian Cultus Minister has bestowed it on a number of *Privatdocenten*—doctors of medicine for the most part—without first consulting the faculty (the body corporate of university teachers). No wonder that a certain amount of discontent is felt in university circles. There is every reason to believe that, had the faculty been asked, they would have advised the promotion to a real professorship in some cases, and in others would have considered the scientific work of the claimants hardly such as could justify the preference.—*Berlin Cor. British Med. Journal.*

When your broach fails to take and wind cotton perfectly, draw it a time or two through a once-folded piece of fine sandpaper.

